# An Account of the Progress of Knowledge Respecting Materia Medica, from June 1818 to January 1819

**Published:** 1819-02

**Authors:** 


### *An Account of the Progress of Knowledge respecting Materia Medica, from June* 1818 *to January* 1819.

"Non enim quidlibet ægris inculcasse; sed cogitasse quid maxime conveniret, et id usu explorasse, quod antè conjectura aliqua dicissent."—CELSUS, lib. i.

PREVIOUSLY to a detail of the subjects we have to treat of in a particular manner, it may be proper to notice in general terms the various systematic works that have recently appeared on materia medica; of which, the most important is the *Paris Pharmacopœia*.* Of this we must speak in terms of warm approbation. The general arrangement of the matter is perspicuous and judicious; the language concise and accurate; and the whole highly creditable to M. HALLE, who superintended the composition.

One of the principles which influenced the labours of the authors is particularly deserving of praise, which they express by observing—"Perspicere præstantius certè est, quam despicere:" this relates to those formula which have been long established, and kept prepared by apothecaries, with the properties of which the public suppose themselves to be acquainted, and, consequently, are in the habit of employing without having recourse to professional advice.

Professor SALVA has published an account of the materia medica employed by him in his practice at the Royal Medical School at Barcelona;† which we notice because of the judicious manner in which he has arranged and described the matters of which it treats.

The history of each medicine is divided into six para-

---

* *Codex Medicamentarius, sive Pharmacopœia Gallica;* editus a Facultate Medica Parisiensi, anno 1818. 4to. pp. 393; apud Hacquart.

† *Tercer año Medico-Clinico de la real Escuela de Medicina Practica de Barcelona,* 4to. 1818.

graphs: the first indicates the name given to the substance by Linnæus, and the work in which it has been treated of by that naturalist; the second describes its physical properties; the third, its chemical qualities; the fourth, its most evident and general medicinal properties; the fifth treats of its curative property, which is the necessary result of the action of the preceding one: this last paragraph has often been left blank, in consequence of the obscurity in which the consecutive effects of medicine are still enveloped. The same reason induced him to arrange them alphabetically, rather than by any systematical classification. The sixth paragraph indicates the mode of using the remedy. Neither in the number of the medicinal substances employed, nor in the composition of the formula which he has described, can Prof. Salva be said to be subject to the censure for too great multiplicity and complexity, which has been passed on many productions of this kind: on the contrary, we find many remedies which we consider as highly useful for their variety, and essentially necessary for their properties, not employed by him; such as arsenic, belladonna, colocynth, digitalis, gentian, scammony, &c.

An amplified and corrected edition of the *system of Materia* of Schwilgue, by the late M. Nysten, has also recently appeared. The merit of this work is so well known and generally acknowledged, that any observations on our part are unnecessary.

A new and improved edition of the excellent pharmaceutic work of Dr. Cadet de Gassicourt, with notes by Dr. Pariset, has also been lately produced.

The physicians of the United States are very actively engaged in the investigation of the medicinal qualities of the plants produced on that part of the globe; several of which are indigenous to it, and highly estimable in the practice of medicine. The most important works on this subject now in the course of publication are those of Dr. Barton* and Dr. Bigelow:† that of the latter is particularly valuable

---

* *Vegetable Materia Medica of the United States, or Medical Botany*, &c.; by W. P. C. Barton, M.D. Professor of Botany in the University of Pennsylvania, &c. &c. Carey and Son, Philadelphia; and Souter, London, 1818.

† *American Medical Botany;* being a Collection of the native Medicinal Plants of the United States, containing their botanical History, chemical Analysis, and properties and uses in Medicine, &c.; by Jacob Bigelow, M.D. Rumford Professor and Lecturer on Materia Medica and Botany in Harvard University. Cummings and Hilliard, Boston; and Souter, London, 1818.

from the great erudition of the author, and his extensive research respecting the observations that may have been made respecting the medical qualities of those plants already generally known, and from the accuracy with which the chemical analysis of them has been conducted. The work of Dr. Barton merits the highest praise for the accuracy of the botanical history, and the manner in which the representations of the plants are executed. We need not notice these works more at length on the present occasion, having already inserted in our Journal some extracts from them, which we shall repeat from time to time, respecting such plants as are known, or appear to be worthy of being brought into use, in Europe.

A new edition of the Dispensatory of Mr. THOMSON has also lately appeared,* which embraces every thing of most importance relative to pharmacy, &c. up to the period of its publication.

We have also to notice a work on the same subject,† by Mr. GRAY. This is chiefly adapted for the use of the chemists of the present period, who have become what apothecaries were formerly.

As the mode of action of numerous materia medica is obscure, and of many of them altogether unknown,—and also from several of them producing various effects when employed under different circumstances,—we shall consider those of which we have to treat in alphabetical order, rather than according to any systematic arrangement founded on their supposed properties.

ANTIMONIUM TARTARIZATUM has been introduced to the notice of physicians by Dr. BALFOUR, as a medicine possessed of properties not generally attributed to it. The author, in his treatise on this subject,‡ observes, that it has been ordinarily employed only in *nauseating* doses, with a view to produce general relaxation and perspiration with-

---

* *The London Dispensatory;* containing the Elements of Pharmacy, the Botanical Description, Natural History, Chemical Analysis, and Medical Properties, of the Substances of the Materia Medica, &c.; by A. T. Thompson. 8vo.

† *A Supplement to the Pharmacopœias;* including not only the Drugs and Compounds which are used by professional or private Practitioners of Medicine, but also those which are sold by Chemists and Herbalists, and for other Purposes; by L. F. Gray. 8vo.

‡ *Observations, with Cases, illustrative of the sedative and febrifuge Powers of Emetic Tartar.* By W. Balfour, M.D. See *London Medical and Physical Journal,* vol. xl. p. 410.

out heating the system; but that its sedative powers, independent of these obvious effects, were not known to the physicians whom he consulted respecting the intentions with which they had employed this remedy. Dr. Balfour has adduced the most ample testimony of its powerful sedative effects, and, consequently, of its great utility in all cases where it is desirable to lessen the action of the animal economy: such testimony as, we hope, will render the use of that medicine for these purposes more generally known.

Arsenicum. The superior efficacy of this to all other known remedies in many cases of intermittent fever, is beginning to be as well ascertained on the continent as it has been for many years in England. Amongst various reports in its favour, we may particularly notice that of M. Gasc, which is deduced from observations made during his official attendance on the hospitals at Dantzick. "The number of patients with fever was so great in the hospital," says that physician, "and the disease had been so rebellious to the ordinary modes of treatment, that I determined to have recourse to the use of arsenic.* I first administered it to some patients affected with quartan fever, selecting those cases which were not complicated with other diseases, and in which the intermission was well marked. My first trials of it were so satisfactory, and the successful results so numerous, that I did not hesitate to extend the use of it to almost all the cases of fever of that species in the hospital, without regard to particular complications of disease, provided they were not of a nature to contra-indicate it in a positive manner. Of seventy-four patients to whom it was given,—sixty-three had quartan, six tertian, and five quotidian or anomalous, fever: forty left the hospital cured within a month; half of the remainder were free from fever at that period; and the others were still under treatment, which had been suspended for various reasons."

Colchicum *autumnale*. The efficacy of this medicine for the relief of gout and rheumatism, and the safety with which it may in general be administered under judicious management,† are now generally known in Europe; and it has been commonly employed in the United States for some time with the same intentions. The diuretic and cathartic property of this medicine led Dr. Clarke, of Philadelphia, to suppose that it would be well adapted for many cases of dropsy; and he has employed it in one case with success.—

* The preparation he employed was similar to the *Liquor Arsenicalis* of the London Pharmacopœia.

† For some useful observations on this subject, see *London Medical and Physical Journal*, vol. xl. p. 396.

"A black woman, aged 32 years, had ascites and anasarca of the lower extremities. I gave her two hundred drops of the tinc. colchici in the morning, which produced a very copious discharge of urine and watery fæces. The use of the medicine was continued for nine days, in which time the dropsical swelling had completely disappeared, and nothing was then left for me but to prescribe a little tonic medicine. In the space of nineteen days, I had the satisfaction of seeing her restored to her usual state of health."*

Galvanism. We shall quote the remarks of Dr. Wilson Philip on the use of this agent as a remedy for disease.

"The experiments related in the preceding enquiry† seem to point out, more precisely than former observations have done, what we are to expect from the use of galvanism in the cure of disease; and I think it will appear from what I am about to say, that, to the want of discrimination in its employment, we must ascribe the little advantage which medicine has hitherto derived from the discovery of this influence.

"It is an inference I have already had occasion to observe, from my own experiments and observations, as well as those of others, particularly of M. le Gallois, that what is called the nervous system comprehends two distinct systems,—the sensorial, and the nervous system properly so called. Now it does not appear, from the foregoing enquiry, that galvanism can perform any of the functions of the sensorial system; yet, in the greater number of instances in which it has been used in medicine, it has been expected to restore the sensorial power: it has been expected to restore hearing, and sight, and voluntary power. It may now and then happen in favourable cases, from the connexion which subsists between the sensorial and nervous systems, that, by rousing the energy of the latter, we may excite the former. It would be easy to show, that we have little reason to expect that this will often happen. We have also reason to believe that galvanism has no other power over the muscular system than that of a stimulus; we are, therefore, to expect little more advantage from it, in diseases depending chiefly on faults of the sanguiferous system, than from other stimuli, &c. But I cannot help regarding it as almost ascertained, that in those diseases in which the derangement is in the nervous power alone, where the sensorial functions are entire, and the vessels healthy, and merely the power of

---

* *American Medical Recorder*, vol. i. p. 377.

† *An Experimental Enquiry into the Laws of the Vital Functions*, &c.; by A. P. Wilson Philip, M.D. F.R.S.E. &c. Second edition. London, 1818.

secretion, which seems immediately to depend on the nervous system, is in fault, galvanism will often prove a valuable means of relief."

Habitual asthma, unaccompanied with evident inflammation, was the species of disease in which it was employed with most benefit. Many of the patients were working people of the city where Dr. Philip resides, who had been obliged to abandon their usual occupation in consequence of it; and some of them, from its long continuance, without any hope of returning to regular work. Most of them had tried the usual means in vain. By the use of galvanism, they were relieved in different degrees, but all sufficiently to be restored to their employments. Several of them, whom Dr. Philip saw lately, said they had continued to work without any inconvenience, although they had not used galvanism for many months. Some, in whom the disease had been wholly removed, remained quite free from it; some have had a return of it, and have derived the same advantage from galvanism as at first. It was applied in the following manner:—

Two thin plates of metal, about two or three inches in diameter, dipped in water, were applied, one to the nape of the neck, the other to the pit of the stomach, or rather lower. The wires from the different ends of the trough were brought into contact with these plates, and as great a galvanic power maintained as the patient could bear without complaint. The operation was discontinued as soon as the patient said that his breathing was easy, which generally happened in from five minutes to a quarter of an hour. It was seldom used more than once a-day; but, in some of the more severe cases, it was applied morning and evening. It failed to give considerable relief only in about one-tenth of the cases to which it was applied.

In order to apply this powerful agent with sufficient precision, and with a confident expectation of beneficial results, not only a correct knowledge of the nature of the individual disease, but accurate anatomical and general physiological information, is requisite; particularly as regards the nervous system. It is also necessary that the medical practitioner be well acquainted with the nature of that agent, its mode of influence, and the various ways of producing and regulating it according to accidental circumstances. There is no work from which this knowledge can be so fully obtained, and with so much facility, as from the treatise of Dr. BOSTOCK on this subject.*

---

* *An Account of the History and present State of Galvanism;* by John Bostock, M.D. F.R.S. &c. pp. 158. London, 1818.

Helleborus, *albus* et *niger*. A Memoir, relating an account of some experiments made with these plants by M. Schabel of Weissenbourg, was read, in September last, to the Medical Society of Emulation of Paris. Although nothing of great importance is adduced, yet we consider every thing relative to this subject too interesting to be passed over without notice. We shall not detail the experiments of M. Schabel, but merely the conclusions he has deduced from them.

The lethiferous properties of those plants very much resemble each other.

These properties appear to reside principally in the resinous parts, and are not neutralized by the tincture of nut-galls, as many physicians have asserted. The emetic properties attributed to the resinous, and the narcotic to the gummy, parts of these roots, has not been confirmed by experience.

The influence of these poisons is not only injurious to animals, but also to vegetables.

The effects they produce on the higher classes of animals are determined by the same circumstances on which those of the poisonous bitters, arsenic, and prussic acid, depend.

Their action is most marked when they are introduced into the blood-vessels, or applied on the serous membranes, or on organs well furnished with blood-vessels. Their influence is exerted through the medium of the circulation.

Their effects are—slow, difficult respiration; slowness, and sometimes irregularity, of the pulse; vomiting of mucous and bilious matter; increased secretion of saliva; vacillation; vertigo; convulsions, followed by tetanus; and diminution of heat. At length the animal becomes cold, respires after long intervals, exhibiting hardly any signs of life, which is lost by degrees.

In those animals who have not been killed immediately by this poison, the lungs are found heavy, gorged with blood, either generally or partially of a brownish colour, and covered with a dense membrane. The gall-bladder is filled with bile, a large quantity of which is found in the superior part of the intestinal canal, and the mucous membrane of this cavity often shows signs of inflammation. The liver is frequently distended with blood. A large quantity of black blood is found in the great venous trunks; and the right, and sometimes the left, cavities of the heart. The irritability of the muscles of both organic and animal life is still considerable, and the nerves possess the power of conveying impressions. The other organs do not evince any thing remarkable.

Black hellebore was much employed, two or three centuries since, as an emenagogue, but it appears to be much neglected at the present time. Dr. MACLEAN, of Edinburgh, is disposed to think very favourably of its powers in that respect, and has published some observations tending to demonstrate them *

HYDRARGYRUS. Dr. SALAMANCA, of the Royal Marine Hospital of Spain, has made public some observations on the use of the prussiate of mercury in the treatment of syphilis, and some diseases of the lymphatic system; apparently owing to the remarks of M. CHAUSSIER respecting the superiority of this preparation for those purposes. The cases related by Dr. Salamanca do not appear to us either to be sufficiently interesting to merit detail, or to prove what the author would indicate. The history of the cases is too concise to permit any satisfactory judgment to be formed respecting them; and we consider that the measures generally adopted by judicious practitioners would have been equally successful. This author, however, states that, besides syphilitic, many dartrous and psoric affections, which had withstood all the usual means, disappeared under the use of this remedy. The ordinary dose was a tea-spoonful of a solution formed of eight grains of the prussiate of mercury in eight ounces of water.

MOXA. We notice this subject principally from a desire to excite the attention of our readers to a remedy that it is much to be wished were introduced into practice in this country. The cases of hydrocephalus, in which it was employed by Dr. REGNAULT, related in the last volume of this Journal, are such testimonials in its favour as cannot fail to make a deep impression on the mind of every reflective medical practitioner; and, we should think, would effect what we have indicated.

NUX VOMICA. The accounts published by MM. FOUQUIER and HUSSON of their experiments with this remedy, have led to an extensive trial of its properties by other physicians; the results of which show it to be an highly valuable medicine, but which requires much caution in its application. Some cases, where it has been used by Drs. LESCURE and FINOT with the most evident benefit, will be detailed in another part of the present number of this Journal. We have chosen those to which we refer, from a series of successful instances of the application of this remedy, from their particularly pointing out its more immediate effects, and the ill consequences that may ensue from its use when not judiciously administered.

TODDALIA. Although the consumption of cinchona bark

---

* See *London Medical and Physical Journal*, vol. xl. p. 16.

at the present period is very small in proportion to what it was half a century since, yet, as MM. Humboldt, Bonpland, and other travellers, assert that it appears probable that the forests of Peru and the Andes will, before a century is passed, be exhausted of that valuable remedy, every substance apparently resembling it in its properties merits attentive consideration. Many physicians think that the bark of the willow, oak, horse-chesnut, &c. in our country, is no mean substitute for that of the loxa of Peru; but it is probable that it is to warmer climates we must look for the most efficacious remedies of this class. Dr. Virey, in a Memoir in the Journal de Pharmacie, states, that M. Bosc has received from M. Hubert, a botanist of the Isle of Bourbon, some specimens of the bark of a shrub, which is generally employed in the East-Indies, the islands of Madagascar, France, Bourbon, &c. as a febrifuge in place of cinchona, with the most satisfactory results.

This bark is rolled somewhat like the cinchona, covered with an epidermis of a brown or greyish colour, interspersed with yellowish spots. The epidermis is about a line in thickness, granular in its tissue, and of a bright yellowish-brown colour: its taste is slightly bitter and aromatic. The interior bark, which constitutes the *liber*, is thin; of a reddish-brown colour; of a singularly bitter and poignant taste, somewhat resembling pepper in warmth, with a mixture of sweetness: its fracture does not present a resinous appearance.

The shrub that furnishes it is very common in Asia and some of the African islands: it is described by Van Rheede, in his *Hortus Malabaricus* (tom. v. fig. 41,) under the name of *Kaka-Toddali*. It is a small thorny shrub, with tortuous branches, according to Commerson. The flowers are axillary, composed of a five-parted calyx, five petals, five stamens, three styles, and three stigmas. The fruit is a berry about the size of a nut, containing five oval-shaped seeds: it is rugous on its surface, and contains a volatile oil, in the same manner as orange-peel. The leaves are ternate, covered like those of milfoil with small translucid points, oval, lanceolated, a little dentated, and armed with prickles like the stalks and branches.

Linnæus placed this plant in the class *pentandria*, order *trigynia*, under the name of *paullinia Asiatica*. Schreiber made it the genus *crantzia*; which has been changed into that of *scopolia* by Smith and Wildenow, under the name of *scopoliata aculeata*; and arranged with the *adelia* by De Lamark. Jussieu preserved the name of *toddalia*, by which it is known on the coast of Malabar.

