# Corrigenda

**Published:** 1819-01

**Authors:** 


					? 112, ? 23, after Materia add Med tea.

				

